# Supplementary material for: Safety assessment of a novel C-type natriuretic peptide derivative and the mechanism of bone- and cartilage-specific toxicity
Source: PLoS One. 2019 Sep 11;14(9):e0218229. doi: 10.1371/journal.pone.0218229 (PMC6738601; doi:10.1371/journal.pone.0218229)
Supplement: S1 Table — (DOC) [file pone.0218229.s001.doc]

Supporting Table 1. Histopathological findings in rats treated subcutaneously with ASB20123 for 4 weeks in study 1
