# Supplementary material for: Safety assessment of a novel C-type natriuretic peptide derivative and the mechanism of bone- and cartilage-specific toxicity
Source: PLoS One. 2019 Sep 11;14(9):e0218229. doi: 10.1371/journal.pone.0218229 (PMC6738601; doi:10.1371/journal.pone.0218229)
Supplement: S3 Table — (DOC) [file pone.0218229.s003.doc]

Supporting Table 3. Histopathological findings of the femur and tibia in rats treated subcutaneously with ASB20123 for 4 weeks followed by 13 weeks recovery period in study 3.

|  |  |  | Group | | | Vehicle |  | ASB20123 | |
| --- | --- | --- | --- | --- | --- | --- | --- | --- | --- |
| Organs/Tissues | | | Dose (mg/kg/day) | | | 0 |  | 0.5 | 5.0 |
|  | Findings |  | No. of animals | | | 5 |  | 5 | 5 |
| Femur (proximal) | | |  |  |  |  |  |  |  |
|  | Degeneration/necrosis, epiphysis/metaphysis | | | | | - |  | 1 (+++) | 2 (+++) |
| Femur (distal) | |  |  |  |  | - |  | - | - |
| Tibia (proximal) | |  |  |  |  | - |  | - | - |
| Tibia (distal) | |  |  |  |  |  |  |  |  |
|  | Deformation of tibia distal end joint* | | |  |  | - |  | 2 (+) | 2 (+) |

Grades: -, normal; +, slight; +++, severe.

The numbers of animals with pathological changes are listed. Vehicle: 0.03 mol/L acetic acid buffer solution (pH 4) containing 10 w/v% sucrose and 1 w/v% benzyl alcohol.

*: with discontinuity of articular surface and minimal focal degeneration/necrosis of cartilage and osseous tissue.
