# Supplementary material for: Safety assessment of a novel C-type natriuretic peptide derivative and the mechanism of bone- and cartilage-specific toxicity
Source: PLoS One. 2019 Sep 11;14(9):e0218229. doi: 10.1371/journal.pone.0218229 (PMC6738601; doi:10.1371/journal.pone.0218229)
Supplement: Attachment — Submitted filename: Response to Reviewers.docx [file pone.0218229.s004.docx]

Dear Professor Bader,

Re: “Safety assessment of a novel C-type natriuretic peptide derivative and the mechanism of bone- and cartilage-specific toxicity” by Takafumi Yotsumoto, Naomi Morozumi, Ryuichi Nakamura, Toshimasa Jindo, Mayumi Furuya, Yasuyuki Abe, Tomonari Nishimura, Hiroaki Maeda, Hiroyuki Ogasawara, Yoshiharu Minamitake, and Kenji Kangawa; ms.no PONE-D-19-14712.

We are most grateful to you and the reviewers for the helpful comments on the original version of our manuscript. We have taken all these comments into account and submit, herewith, revised version of our manuscript.

We have addressed journal requirement and all the comments by the academic editor and reviewers #1 and #2, as indicated on the attached pages, and we hope that the explanations and revisions of our work are satisfactory.

We hope that the revised version of our manuscript is now suitable for publication in the PLOS ONE and we look forward to hearing from you at your earliest convenience.

Yours sincerely,

Yotsumoto Takafumi, M.S.

Journal requirement

When submitting your revision, we need you to address these additional requirements.

Please ensure that your manuscript meets PLOS ONE's style requirements, including those for file naming. The PLOS ONE style templates can be found at
<http://www.journals.plos.org/plosone/s/file?id=wjVg/PLOSOne_formatting_sample_main_body.pdf> and <http://www.journals.plos.org/plosone/s/file?id=ba62/PLOSOne_formatting_sample_title_authors_affiliations.pdf>

Response.

According to journal requirement, we have checked our revised manuscript to meet PLOS ONE’s style requirements.

At this time, we request that you  please report additional details in your Methods section regarding animal care, as per our editorial guidelines: 1) Please provide details of animal welfare (e.g., shelter, food, water, environmental enrichment) 2) please describe any steps taken to minimize animal suffering and distress, such as by administering analgesics, 3) please include the method of sacrifice and 4) Please describe the care received by the animals, including the frequency of monitoring and the criteria used to assess animal health and well-being. Thank you for your attention to these requests.

Response.

According to journal requirement, we revised the methods section about animal care. Details of animal welfare (e.g., shelter, food, water, environmental enrichment) were mentioned in line 88-92, the method of sacrifice was in line 92-94, and frequency of monitoring was in line 132-133 in the Revised Manuscript with Track Changes, respectively. The steps taken to minimize animal suffering and distress and the criteria used to assess animal health and wellbeing were described in the Guidelines for Animal Experiments of LSI Medience Corporation and/or Asubio Pharma Co., Ltd, and these studies were conducted in accordance with these guidelines.

We are grateful to the Reviewer #1 for the useful comments and suggestions that have helped us to improve our manuscript considerably. As indicated in the following responses, we have taken all your comments and suggestions into account in our revised manuscript. The line numbers appearing in the following responses indicate those of the text in the “Revised Manuscript with Track Changes” file.

Comments by reviewer #1.

Comment 1.

Introduction, line 44:- replace “expecting” with “exciting” This line should read “…(CNP) analog is one of the most exciting therapeutic …”.

Response.

Thank you for your comment. We replaced from “expecting” to “exciting” in the revised version of our manuscript. Please confirm the revised sentence in line 44 in the Revised Manuscript with Track Changes.

Comment 2.
The reviewer suggest more background information regarding the rational for using a CNP/ghrelin chimeric peptide should be provided eg describe the potential for interaction with hyaluronic acid.

Response.

Thank you for your valuable suggestion. We added the following sentence about the background information of ASB20123 in line 55-60 of the Revised Manuscript with Track Changes;

“The application of C-terminal part of ghrelin resulted in the higher stability of CNP analogs, compared to that of CNP-22 as the native form; it also improved their bioactivity as stimulators of endochondral bone growth. Furthermore, the C-terminal part of the ghrelin includes a BX7B motif, the cluster of basic amino acids might be implicated in the interaction with hyaluronic acid molecules which are the components of extracellular matrix in growth plate.”

In addition, we added 4 references in this part and it is shown in line 400-412 in the Revised Manuscript with Track Changes.

Comment 3.
Page 12, line 191
There is no mention in the results section of any differences in blood chemistry measurements observed between the groups eg triglycerides (mentioned in the methods section). If there were no significant differences, the reviewer suggests line 191 could read “… ophthalmology, blood chemistry, urinalysis …”.

Response.

Thank you for pointing out. We missed describing the change of CPK activity. We added in line 201, 204-205 and 212 of the result section, in line 328-330 of the discussion section in the Revised Manuscript with Track Changes.

Comment 4.
It would be helpful for the reader if the age of the rats was included in the title of Supporting Table 1 or alternatively the title to the table read “Study 1 – histopathology in rats treated subcutaneously with …”.

Response.

Thank you for your comment. We added the study No. in the title of all figures and supporting table 1.

We are grateful to the Reviewer #2 for the critical comments and useful suggestions that have helped us to improve our manuscript considerably. As indicated in the following responses, we have taken all your comments and suggestions into account in our revised manuscript. The line numbers appearing in the following responses indicate those of the text in the “Revised Manuscript with Track Changes” file.

Comments by reviewer #2.

Major Comment.

Figure 4 of this manuscript shows a very interesting inverse relationship in response to ASB20123 infusion with the drug increasing bone length and decreasing density in both the cortical and trabecular bone. If this is the case, then it may limit the use of the drug because while it increases long bone length, it also results in less bone mineral density, which is unfavorable.

It would be very interesting to see if the BMD increases with time after the initial CNP analog infusion period has ended. In other words, if ASB20123 increases bone length, does it always also decrease bone mineral density or does it return to normal values after the ASB20123 treatment is discontinued. To test this, the investigators should analyze another group of animals 6 weeks after the last ASB20123 injection and determine if BMD is still reduced or whether it increased in the absence of the drug. The question being addressed with this experiment is whether any increase in bone length resulting from ASB20123 infusion also results in permanent reductions in BMD? If the later scenario is observed, then this could limit the utility of ASB20123 in the treatment of achondroplasia if it always results in less dense, and presumably, weaker bone.

Response.

Thank you for your valuable suggestion. It is very important to clarify the relationship the reduction of BMD and elongation of bone length caused by ASB20123 treatment. It is especially needed to care of the decrease of BMD in the treatment of achondroplasia, because the lower BMD in achondroplasia population was reported (Sims D, et al. Whole-body and segmental analysis of body composition in adult males with achondroplasia using dual X-ray absorptiometry. PLos One, 14(3): e0213806, 2019).

In this study, thickness of epiphyseal plate of femur, BMD and bone related toxicity were observed in the rats treated with ASB20123 at dose of 0.5 mg/kg/day and more for 4-week. Meanwhile, in a previous study, the body length increased significantly after continuous *sc* infusion at dose of 0.05 mg/kg/day and more for 12-week (Morozumi N, et al. ASB20123: A novel C-type natriuretic peptide derivatives for the treatment of growth failure and dwarfism. PLos One, 14(2): e0212680, 2019). At the same time, the BMD decreased significantly at dose of 0.15 mg/kg/day and more (internal data). We consider that these phenomena were caused mainly by rapid and excessive induction of bone growth in normal juvenile animals.

In addition, we evaluated the recovery of BMD and bone toxicity after 13-week recovery period. These results described in line 262-277 of result section and line 352-364 of discussion section in the revised version of our manuscript and added Fig. 5. The decreased BMD values induced by ASB20123 treatment did not return to the value of control group after 13-week recovery period. However, the BMD values in the ASB20123 treated groups increased similarly to those in the control group for 13 weeks. These results indicate that at least further reduction in BMD could be prevented by discontinuation of treatment.

Considering these results, the dosage regimen of CNP derivative would be a very important key factor for the success as a therapeutic drug.

Minor Comment.

The manuscript would benefit by editing from a native English-speaking person. Trabecular bone is the newer and more common term for “spongy” bone.

Response.

Thank you for pointing out. We replaced from “spongy” to “trabecular” in the revised version of our manuscript. Also, a native English-speaking person in our company have checked our revised manuscript.
